# Exploring the Prospects of Engineered Newcastle Disease Virus in Modern Vaccinology

**DOI:** 10.3390/v12040451

**Published:** 2020-04-16

**Authors:** Muhammad Bashir Bello, Khatijah Yusoff, Aini Ideris, Mohd Hair-Bejo, Abdurrahman Hassan Jibril, Ben P. H. Peeters, Abdul Rahman Omar

**Affiliations:** 1 Department of Veterinary Microbiology, Faculty of Veterinary Medicine, Usmanu Danfodiyo University PMB, Sokoto 2346, Nigeria; bbtambuwal@gmail.com; 2 Laboratory of Vaccines and Immunotherapeutics, Institute of Bioscience, Universiti Putra Malaysia, Serdang, Selangor 43400, Malaysia; kyusoff@upm.edu.my (K.Y.); aiini@upm.edu.my (A.I.); mdhair@upm.edu.my (M.H.-B.); 3 Department of Microbiology, Faculty of Biotechnology and Biomolecular Sciences, Universiti Putra Malaysia, Serdang, Selangor 43400, Malaysia; 4 Department of Veterinary Clinical Studies, Faculty of Veterinary Medicine, Universiti Putra Malaysia Serdang, Selangor 43400, Malaysia; 5 Department of Veterinary Pathology and Microbiology, Faculty of Veterinary Medicine, Universiti Putra Malaysia Serdang, Selangor 43400, Malaysia; 6 Department of Veterinary Public Health and Preventive Medicine, Faculty of Veterinary Medicine, Usmanu Danfodiyo University PMB, Sokoto 2346, Nigeria; jibrilah50@yahoo.com; 7 Department of Virology, Wageningen Bioveterinary Research, POB 65, NL8200 Lelystad, The Netherlands; ben.peeters@wur.nl

**Keywords:** Newcastle disease virus, reverse genetics, vaccines, infectious diseases, cancer

## Abstract

Many traditional vaccines have proven to be incapable of controlling newly emerging infectious diseases. They have also achieved limited success in the fight against a variety of human cancers. Thus, innovative vaccine strategies are highly needed to overcome the global burden of these diseases. Advances in molecular biology and reverse genetics have completely restructured the concept of vaccinology, leading to the emergence of state-of-the-art technologies for vaccine design, development and delivery. Among these modern vaccine technologies are the recombinant viral vectored vaccines, which are known for their incredible specificity in antigen delivery as well as the induction of robust immune responses in the vaccinated hosts. Although a number of viruses have been used as vaccine vectors, genetically engineered Newcastle disease virus (NDV) possesses some useful attributes that make it a preferable candidate for vectoring vaccine antigens. Here, we review the molecular biology of NDV and discuss the reverse genetics approaches used to engineer the virus into an efficient vaccine vector. We then discuss the prospects of the engineered virus as an efficient vehicle of vaccines against cancer and several infectious diseases of man and animals.

## 1. Introduction

Vaccines are undoubtedly among the most effective fighters of infectious diseases. They have historically been used to completely eradicate or at least substantially reduce the menace of many human and animal diseases [1,2,3]. Conventional vaccines can be broadly classified into two groups. The first group includes the live attenuated vaccines that are highly effective due to their ability to induce immune responses that are essentially similar to those due to natural infection [4,5]. Unfortunately these vaccines often retain the tendency of reversion back to virulence. The second group includes the inactivated vaccines, which are known to be incredibly safe as a result of their non-replicating nature, but are often poorly immunogenic and therefore, do not elicit a long lasting immunity [6]. Furthermore, both live attenuated and inactivated vaccines have failed to effectively curb the menace of a variety of major global pathogens. These limitations altogether quest for the need to develop novel vaccine strategies that could potentially overcome the weaknesses of the conventional vaccines. Interestingly, the recent advancements in molecular genetics and bioinformatics have paved way for the emergence of next generation vaccine technologies such as synthetic peptides, DNA vaccines, recombinant viral-vectored vaccines, and reverse genetics-based vaccines, which are all currently on the path of revolutionizing medical and veterinary vaccinology [7,8]. 

Newcastle disease virus is one of the most important avian viral pathogens that inflict huge economic losses in the global poultry industry [9]. The virus is highly genetically diverse, with currently more than 20 phylogenetically distinct genotypes based on the recently proposed NDV taxonomy criteria [10]. Following the recovery of the first strain of the virus by reverse genetics 20 years ago [11], tremendous progress has been recorded in the genetic manipulation of various strains of the virus. So far, NDV has been engineered to express rationally designed, safe and highly stable protective antigens against several human and animal pathogens (Table 1). The virus has also been genetically reprogrammed for improved oncolytic efficacy against a variety of human cancers (Table 2). Thus, the prospects of genetically engineered NDV in the era of modern vaccinology cannot be over emphasized. In this review, we start by describing the molecular biology of NDV and the various approaches used in the genetic manipulation of the virus for effective vaccine delivery. We then discuss the potentials of the engineered virus as a vaccine against cancer and other life threatening diseases in man and against economically important viruses in various domestic animals. 

## 2. Newcastle Disease Virus

### 2.1. Architecture and Genome Organisation

Ultrastructurally, the particles of NDV are pleomorphic in shape with diameters ranging from 100–500nm. They essentially consist of the ribonucleoprotein (RNP) surrounded by the viral envelop with its surface glycoproteins that project as spikes. The RNP is made up of the RNA genome completely encapsidated by a protein called nucleocapsid protein (NP). Other proteins associated with the RNP include the large protein (L), which is the RNA-dependent RNA polymerase and its co-factor, the phosphoprotein (P) [12]. Together, they form a helical structure surrounded by a lipid bilayer envelop with surface projections of hemagglutinin-neuraminidase (HN) and fusion proteins (F). The matrix protein (M) is found just beneath the viral envelop and maintains the shape and structure of the virion [13].

The genome of NDV is either 15,198, 15,192 or 15,186 bp in size [14]. It is a single stranded, non-segmented negative sense RNA that consists of leader (55 nucleotides) and trailer (114 nucleotides) terminal sequences separated by six genes in the order 3′-NP-P-M-F-HN-L-5′. These terminal sequences are highly conserved across most of the paramyxoviruses [12,15] and house the regulatory signals for virus replication [16]. In addition, each gene in the NDV genome encodes a single protein and is characterized by having a coding sequence flanked by highly conserved gene start (GS) and a gene end (GE) transcriptional signals [17]. These features of NDV genome are shared by most of the paramyxoviruses, suggesting a common transcription and replication strategy.

### 2.2. Virulence Determinants

Using reverse genetics, RNP associated viral structural proteins (NP, P and L) have been shown to collectively contribute to NDV virulence. Experiments involving the swapping of NP between pathogenically different strains revealed a change in virulence of the generated recombinant virus only when NP along with its other replication proteins partners (P and L) were swapped together [18]. Notably, the NP encapsidates the entire genomic RNA while the L functions as an RNA-dependent RNA polymerase that binds to P protein and forms a complex that recognizes the RNP for initiating the process of transcription and replication [19]. Recent evidence has indicated that the L protein can significantly contribute to NDV virulence through its role in virus replication [20]. However, specific regions within the L protein responsible for determining NDV virulence are yet to be discovered. The F protein is the major determinant of NDV virulence. It is synthesized in an inactive form, F_0_, and then becomes activated following its proteolytic cleavage into F1 and F2. The enzyme involved in the cleavage is determined by the amino acid composition of the cleavage site, which in turn varies in virulent and avirulent NDV strains [21]. Avirulent pathotypes generally have a monobasic F cleavage site acted upon by extracellular trypsin like proteases. On the other hand, the virulent strains have multiple basic amino acid residues at their cleavage sites that are activated by intracellular proteases of the furin family [22]. In addition to the cleavage site’s amino acid composition, the sites of post translational modifications, notably glycosylation sites, may play a significant role in the virus virulence. Recently, [23] observed an increase in virus virulence following deletion of N glycans from the heptad repeats of the NDV F protein. Furthermore, [24] showed a dramatic increase in NDV virulence when the cytoplasmic tail from a mesogenic strain was used to replace its counterparts in another lentogenic strain, suggesting the possible role of the F protein’s cytoplasmic domain in determining the virulence of NDV. The HN protein is also believed to be a determinant of NDV virulence. Depending on the location of the termination codon, the HN protein has several subtypes of varying length, with most of the avirulent strains encoding the longest protein made up of 616 amino acids. In contrast, the virulent strains encode the shortest HN proteins of about 571 amino acids. Lentogenic strains such as LaSota and B1 encode an HN protein of 577 amino acids in length [25]. Other lengths of HN protein reported include 572, 580, 582, and 585 amino acids. To investigate whether the HN length diversity is directly associated with the virus virulence, several NDV chimeras with shortened or extended HN length were generated by reverse genetics and tested for pathogenicity using standard procedures. It was revealed that varying the lengths of HN directly affects the functions related to viral replication but not pathogenicity [26]. V protein, a non-structural protein encoded by the p gene, also plays a vital role in determining NDV virulence. The role of V protein is clearly manifested during viral pathogenesis as an interferon antagonist, by selectively targeting STAT-1 for degradation [27]. Recombinant NDVs that cannot express V protein often show no evidence of growth in 9–10 days chicken embryonated eggs and a very impaired growth in cell culture. Those recombinant viruses also manifest sensitivity to exogenous interferon administration, indicating that the V protein plays a role in NDV replication and virulence [27]. Collectively, virulence of NDV is determined by individual or collective roles of both structural and non- structural viral proteins.

### 2.3. Transcription and Replication

The infectious cycle of NDV follows the same pattern as other members of the family *Paramyxoviridae* obviously due to the common genomic features shared among these viruses [28,29]. The viral polymerase complex first recognizes a single promoter located in the 3′ leader sequence of the genome and then moves towards the 5′ end by responding to the conserved gene start (GS) and gene end (GE) signals located at the beginning and end of each gene respectively, to produce various mRNAs [30]. Upon arrival at the GE, the polymerase complex terminates transcription, scans through the intergenic region, and then starts transcription of the next gene and continues in that order until it transcribes the last gene, from where it finally dissociates from the RNA [31]. As the transcription progresses, the synthesized mRNAs are translated into viral proteins whose accumulation in the cell causes the viral polymerase to switch from transcription to replication of the entire viral genome. Further details of the NDV replication cycle have been discussed elsewhere [32]. Noteworthy, efficient replication of NDV can only occur when the genomic length of the virus size is in a multiple of six. This phenomenon is referred to “rule of six” and occurs because during replication, the NP of most paramyxoviruses is associated with exactly six nucleotides on the genomic RNA [33]. Therefore, in any experiment involving the genetic manipulation of NDV, the rule of six must be strictly obeyed [30]. 

## 3. Reverse Genetics System

Reverse genetics is the term used to describe the recovery of recombinant viruses from their cloned cDNA [34]. Viruses generated by reverse genetics can be engineered to either encode desired mutations in the indigenous viral genes or express heterologous antigens as additional proteins. Reverse genetics is therefore a state-of-the-art recombinant DNA technology with considerable impact in modern vaccine design, development and delivery. The first group of viruses known to be amenable to reverse genetics were the positive sense RNA viruses [35] whose genetic material has the same polarity as cellular mRNA and therefore can directly act as templates for protein synthesis. However, owing to peculiar complexities and challenges associated with the replication of negative sense RNA viruses, reverse genetics could not be immediately applied to manipulate those viruses [31] until in 1994 when rabies virus became the first negative sense RNA virus to be successfully rescued from its cloned cDNA [36]. Subsequently, reverse genetics system was established in other negative sense viruses and in 1999, the rescue of the first Newcastle disease virus (NDV) strain entirely from its cloned cDNA was accomplished [11].

### 3.1. Recovery of Recombinant NDV

**Figure 1:**
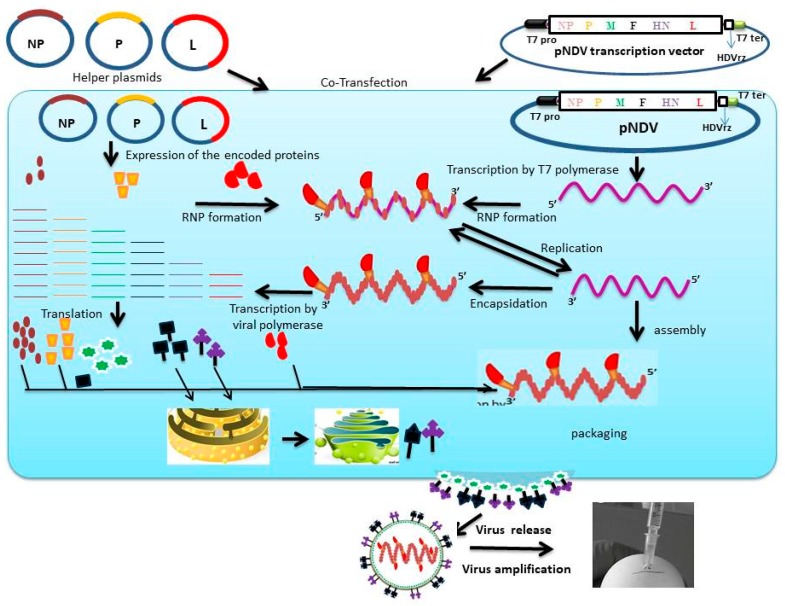
Reverse genetics approach for the generation of engineered Newcastle disease virus in BSR-T7 cells. Full length NDV antigenome flanked by T7 promoter (T7 pro), and autocatalytic hepatitis delta virus ribozyme (HDVrz) is cloned into the transcription vector to form pNDV. Helper plasmids (expression constructs for NP, P and L) are co-transfected with pNDV into BSR-T7 cells, which constitutively express T7 RNA polymerase. Within the cytoplasm, the T7 polymerase transcribes the viral RNA using the pNDV as a template. The helper plasmids express the encoded proteins for association with the transcribed RNA to form the ribonucleoprotein (RNP) template for onward replication cycles. The viral polymerase gradiently transcribes the viral genome into respective mRNAs, which are subsequently translated into proteins. NP, P and L proteins assemble with the newly synthesized negative sense viral RNA while the F and HN proteins are post-translationally modified in the endoplasmic reticulum and Golgi apparatus before being transported to the cell surface. The final event is the release of the recombinant virus by budding, which is then amplified in specific pathogen-free chicken embryonated eggs.

The constructs needed for the recovery of recombinant NDV are helper plasmids and a full length cDNA clone. The helper plasmids are eukaryotic expression vectors that encode the minimum molecular machinery (NP, P and L genes) for the transcription and replication of NDV [37]. On the other hand, the full length cDNA clone represents the entire antigenome of NDV vectored by a transcription vector usually under the control of a T7 promoter. To rescue a recombinant NDV, the full length cDNA construct and the helper plasmids are co-delivered at an optimized ratio, into cells expressing T7 RNA polymerase for the initiation of virus infectious cycle [38]. Commonly used cells in NDV recovery are human epitheloid carcinoma (Hep-2) cells infected with modified vaccinia virus expressing T7 RNA polymerase and genetically engineered Baby Hamster kidney cells (BST-T7) that constitutively express the T7 polymerase. Immediately after transfection, viral RNA will be transcribed from the full length construct and the proteins expressed from helper plasmids will associate to produce the RNP template. Unfortunately, this particular step is largely inefficient and, therefore, the most rate-limiting step in NDV reverse genetics [32]. Once RNP is intracellularly organized, the virus infectious life cycle becomes activated. In a nut shell, the success of generating an infectious virus from a cloned full length cDNA of NDV requires the precise transcription of the full length construct into a viral RNA; optimal co-expression of the NP, P and L genes at a level sufficient to kick-start the replication process; as well as the expression of other viral genes for onward progression of the replication process and the release of the assembled viral particles (Figure 1).

### 3.2. Recent Improvement in NDV Rescue System

The difficulties associated with NDV reverse genetics are largely due to the need to co-transfect at least four plasmid constructs into the same cell. In order to enhance the transfection efficiency, [39] developed a two-plasmid reverse genetics system that dramatically improved the rescue efficiency of NDV. When compared with the conventional four plasmid system, the newly developed system appeared to be superior, enabling an earlier and increased amount of the recovered viruses. In some cases, viruses that could not be rescued using the traditional system were successfully recovered with the aid of this improved reverse genetics system. More recently, a single plasmid-based NDV reverse genetics system was developed [40]. In this approach, the full length construct under the control of T7 promoter was designed to have additional T7 promoter sequences upstream of the GS of P and L genes. Thus, when the plasmid was transfected into cells earlier infected with a fowl pox virus expressing T7 RNA polymerase, a full length viral RNA and two subgenomic RNAs were transcribed. Arguably, some of the subgenomic and full length viral RNAs might be capped and polyadenylated, respectively, at 5′ and 3′ ends by some of the fowl pox enzymes. The mRNAs are in turn translated by the cellular protein synthesis machinery into NP, P and L proteins. Therefore, mere delivery of this single plasmid into cells infected with FP-T7 leads to the assembly of RNP intracytoplasmically, and the eventual production of infectious virus particle. To substantiate the point that the fowl pox enzymes were responsible for the capping and polyadenylation of the subgenomic RNAs, the same plasmid construct was transfected into BSR cells constitutively expressing T7 polymerase and no virus was rescued. Thus, this technique provides an improvement in NDV rescue efficiency and may have important applications in future NDV reverse genetics experiments [40].

### 3.3. Strategies of Foreign Gene Expression Using NDV Vector

Similar to other paramyxoviruses, NDV has been shown to tolerate the insertion of one or more additional genes into its genome without compromising the biological features of the virus. Traditionally, those foreign genes (FG) are inserted as independent transcription units (ITU) made up of GE, IR, GS, and Kozak sequences followed by the coding region of the gene of interest (GOI) [41]. If the foreign antigens are required to be displayed on the surface of NDV, it is important that they are fused with the cytoplasmic and transmembrane domains of the NDV F protein [42]. Noteworthy, the expression level of the FG is considerably dependent on its genomic location in the NDV backbone. Recently, the optimal site for FG expression has been shown to be the P-M junction, which yields the strongest expression signals compared to all other locations in the NDV genome [43]. Contrastingly, the sequential transcription phenomenon states that the closer the gene is to the 3′ end of the genome, the higher the level of its expression [44]. Given that the P-M junction is not located at the extreme 3′ end of the NDV genome, it is logical to ask the question, what exactly is responsible for the strongest level of gene expression at this genomic site? Previous studies have shown that efficient replication of paramyxoviruses requires an optimal NP:P ratio [28]. Therefore, inserting additional gene at locations upstream of the P gene in the NDV genome might affect the relative abundance of the downstream proteins leading to the disruption of the NP:P ratio, which ultimately affects viral replication. On the other hand, when additional genes are expressed at gene borders downstream of the P gene, this NP:P ratio remains unaffected [43]. This probably explains the choice of P-M as the optimal site for FG expression.

**Figure 2:**
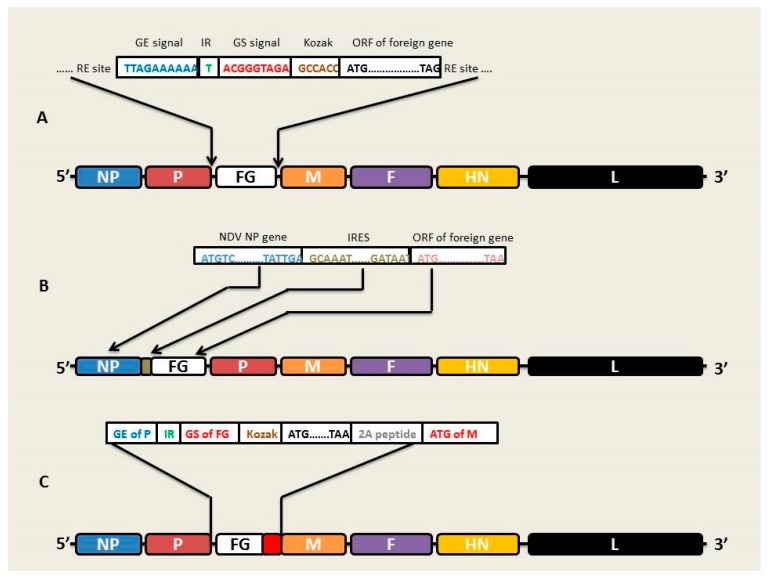
Strategies for foreign gene (FG) expression using NDV as a vector. (**A**) Expression of a foreign gene (FG) as an additional transcription unit. The FG along with NDV transcriptional signals and a Kozak sequence is cloned in a non-coding region, preferably between the P and M genes. (**B**) IRES-mediated expression of an FG. An IRES sequence is placed in between the coding regions of any NDV gene and the FG. (**C**) Expression of an FG via 2A peptide-mediated fusion with any NDV gene.

Another means of FG expression is via an internal ribosome entry site (IRES), which allows the expression of two genes from a single mRNA transcript [45]. In this system, the IRES sequence is inserted immediately downstream of the stop codon of any gene in the NDV backbone followed by the coding region of the gene of interest (Figure 2). During transcription, the two genes separated by the IRES sequence are transcribed into a single mRNA such that the first gene is translated using the default cap-dependent translational machinery while the translation of the downstream gene is cap-independent [46]. The advantage of this system is that the level of gene expression can be regulated by taking advantage of the sequential transcription mechanism of NDV. Thus, when the foreign gene is an immunogen for which a high level of gene expression is needed, the IRES sequence can be inserted immediately downstream the NP gene, followed by the gene of interest. On the other hand, if the FG is a proinflammatory cytokine used in cancer immunotherapy, the IRES and the foreign gene can be located downstream of the M gene so that its expression can be moderate to avoid cytokine storms [47]. Using this strategy, [48] generated a chimeric NDV expressing the F and G proteins of avian paramyxovirus type C as a potential vaccine candidate for turkeys (Table 1). 

In another strategy, NDV has been engineered to express FG via a novel integrating transcriptional unit [72]. In this system, a fusion sequence made up of the foreign gene and 2AUbi is inserted immediately upstream of the translation start codon for any gene in the NDV backbone (Figure 2). The 2AUbi is actually a 94 amino acids sequence made up of the self-cleaving foot and mouth disease virus 2A peptide and ubiquitin coding sequences [73]. Therefore, the FG is expressed as a fusion protein with the downstream protein, which is post-translationally separated by the self-cleaving activity of 2A peptide in vivo. When compared with the system utilizing the independent transcriptional unit (ITU), this system was shown to have higher efficiency of expression. So far, expression of FG via an integrating transcriptional unit has only been demonstrated for NP, M and L genes in the NDV back bone. Preliminary results indicate that M gene yields the optimal result using this system of FG expression [72]. 

In summary, various approaches are available to express FG using the NDV backbone, providing room for a variety of choices depending on the level and manner of gene expression desired. The ITU system is by far the most popular and frequently utilized method of foreign gene expression using NDV vector. Other methods recently evolving include the IRES system as well the integrating transcriptional unit systems. While in the ITU and integrating transcriptional unit systems, the optimal site for foreign gene expression is the P-M junction, with the latter being immediately upstream of the M gene start codon; in the IRES mediated expression, the NP-P junction yields the highest level of gene expression. However, further research is needed to identify the level of foreign gene expression as a fusion protein particularly with the P, F and HN genes of NDV, to verify the claim that expression is highest when the foreign gene is located immediately upstream of the M initiation codon. This will definitely have practical implications for the design of recombinant NDV vectored vaccines.

## 4. Engineered NDV as a Vaccine Vector against Infectious Diseases

### 4.1. Unique Attributes of NDV as a Vaccine Vector

One of the most attractive applications of reverse genetics technology is in the manipulation of viral RNA genomes to express vaccine antigens against several human and animal pathogens [74]. While a number of viruses have been used as vaccine vectors, the efficiency of gene delivery by NDV in both human and animals is unparalleled, due to some unique architectural features of the viral genome. In the first place, the genome of NDV, unlike those of pox and herpes viruses, is simple and encodes only a few structural proteins, which reduces the number of proteins expressed along with the FG and, therefore, enhances the specific immune response against the expressed heterologous proteins [19]. Secondly, each gene has conserved GS and GE sequences, respectively, at its 3′ and 5′ ends, which implies that foreign antigens can be efficiently expressed in the same manner as indigenous NDV proteins, when flanked with those transcriptional signals [32]. In addition, the entire replication cycle of NDV takes place in the cytoplasm, thereby avoiding the risk of random integration of the viral genome into the host cell DNA [16]. These useful properties collectively make NDV an efficient vector for vaccine delivery in different host species.

### 4.2. Engineered NDV as a Bivalent Vaccine in Poultry

To use a recombinant NDV as a bivalent vaccine in poultry, it is imperative that the backbone virus is either a naturally attenuated strain such as LaSota and Hitchner B1 strains, or a genetically engineered NDV encoding some attenuation-inducing mutations particularly at the F protein cleavage site, which is the major determinant of NDV virulence. Although any lentogenic strain of NDV could serve as a bivalent vaccine in poultry, the use of NDV strain more closely related to the circulating field NDV strains stands to offer better efficacy especially in terms of reduction of virus shedding post challenge [8]. Thus, the current trend in ND control focuses on the use of reverse genetics tools to generate the so-called genotype-matched live attenuated NDV vaccines [75,76,77]. Such genotype-matched vaccines, when used as vaccine vectors for other poultry diseases, have the potential to excellently protect against both diseases. The following are examples of diseases in poultry against which NDV has been used to successfully deliver vaccine antigens.

Highly pathogenic avian Influenza is arguably the most fatal viral disease in the global poultry industry. It is caused by avian influenza virus (AIV) of the genus *Influenzavirus* A in the family orthomyxoviridae [78]. The virus has a negative-sense, segmented single-stranded RNA genome encoding at least 10 proteins: polymerase basic 1 (PB1), polymerase basic 1 (PB2), polymerase acid (PA), hemagglutinin (HA), nucleoprotein (NP), neuraminidase (NA), matrix 1 (M1), matrix 2 (M2), nonstructural 1 (NS1), and nonstructural 2 (NS2) [79]. Among these proteins, the HA and NA are the most important inducers of neutralizing immunity [80]. Despite the availability of conventional vaccines, outbreaks due H5NI and H7N7 AIV subtypes have dramatically increased especially in the last two decades. The inactivated AIV vaccines are not effective as they provide suboptimal protection against the disease. The live attenuated vaccines may be highly immunogenic but are not recommended by the OIE due to the potential for novel subtype emergence as a result of genetic reassortment [80]. Thus, alternative vaccine platforms must be explored to control the menace of this fatal avian pathogen. Recombinant viral vectors such as turkey herpesvirus, fowlpox virus, adenovirus, infectious laryngotracheitis (ILT) virus, and Marek’s disease virus (MDV) have all demonstrated promising AIV vaccine delivery efficiency [81]. Similarly, recombinant NDV expressing H5 or H7 of AIV has severally been shown to demonstrate excellent protective efficacy against challenge with both virulent NDV and HPAI virus in chicken [56,82,83]. Details of the protective efficacy of NDV vectored AIV vaccines have recently been reviewed else-where [57].

Infectious bronchitis, an economically important disease of poultry caused by a rapidly evolving coronavirus known as avian infectious bronchitis virus (IBV), has been incriminated in crippling the productivity of the poultry industry all over the world [84]. Current control strategies for the disease rely on the use of traditional live and inactivated vaccines that may not cross protect against other heterologous serotypes [85], necessitating the need to develop vaccines based on the prevailing regionally important serotypes. Since field IBV strains are difficult to attenuate [86], the use of recombinant live vaccines could serve as an attractive alternative in the control of infectious bronchitis in chicken. Unfortunately, due to their large genomes, recovery of recombinant IBV strains by reverse genetics can be highly cumbersome [87]. However, since several studies have shown that most of the neutralizing epitopes against IBV are found in the S1 protein, recombinant viral vectors expressing the IBV S1 protein have the potential to induce protective immune responses in vaccinated chickens. Recently, recombinant NDV strain LaSota engineered to express LX4 type IBV S1 protein has been shown to induce strong cellular and humoral immunity, which provides complete protection against both virulent NDV and LX4 type IBV challenge [54]. Indeed, after vaccination with this bivalent vaccine and subsequent challenge with a virulent IBV field isolate, a marked reduction in the replication of the challenge virus in the trachea was observed especially after booster vaccination [54]. More recently, engineered NDV expressing IBV complete S, S1 or S2 proteins was shown to completely protect against both virulent NDV and IBV challenge, with the highest protective efficacy demonstrated by rNDV expressing complete IBV S protein [42]. Similarly, Abozeid, H.H. et al. ([88]) showed that recombinant NDV expressing codon-optimized S glycoprotein of the Egyptian IBV variant strain IBV/Ck/EG/CU/4/2014 provided excellent protection against both NDV and IBV challenge in chicken. Thus, rNDV is an efficient vector for the delivery of IBV-specific immunogens in chickens.

Another important avian pathogen threatening the global poultry production is infectious bursal disease virus (IBDV). The virus specifically replicates in the chicken’s bursa of Fabricius, resulting in the destruction of the developing B lymphocytes, leading to immunosuppression and enhanced vulnerability to several infectious diseases [89]. Despite the availability of vaccines, control of IBD is still a problem owing to the emergence of antigenically distinct variants as a result of the use of the current IBD vaccines [90]. These variants evade neutralizing antibodies raised against the classical IBDV vaccines, making the control of the disease using the currently available vaccines unreliable. Therefore, to efficiently control this immunosuppressive disease of chicken, there is the need to rationally design vaccines that do not lead to the emergence of novel antigenic variants of the field virus [91]. Since several studies have identified the IBDV VP2 protein to contain the major immunoprotective epitopes against the virus [92], recombinant vaccines based on VP2 protein stand to outperform the currently used IBD vaccines. Interestingly, genetically modified NDV engineered to express IBDV VP2 antigen upstream of the NP gene in the NDV backbone was shown to induce 90% protection against both virulent ND and IBD challenges. More so, a booster vaccination using this bivalent vaccine led to 100% protection against both diseases [58]. This clearly demonstrates the potential of NDV as a promising vector for the delivery of IBDV immunogens in poultry. 

Turkey rhinotracheitis affects the upper respiratory tract of young turkeys leading to high economic losses especially when superimposed with secondary bacterial infection. The disease is caused by avian metapneumovirus (AMPV) subtypes A, B, C, and D, with A and B subtypes being the most widely distributed [93]. Although effective live attenuated vaccines have been developed against this disease, their reversion back to virulence is of serious concern to poultry farmers all over the world. For instance, live vaccines based on AMPV subtypes A and B were shown to acquire a few mutations and regain virulence in turkeys, causing clinical disease similar in severity to the original field strain [94]. Thus, effective control of the disease begs the development of an effective and stable vaccine with no risk of reversion. The surface glycoproteins (F and G) of AMPV are known to participate not only in pathogenicity but also in the induction of neutralizing immunity in the vaccinated host. With the advent of recombinant DNA techniques, viruses vectoring AMPV F or G have been generated and shown to demonstrate some level of protection against turkey rhinotracheitis [71]. More recently, vaccination of turkeys with a recombinant NDV expressing the F and G proteins of AMPV type C (APMV-C) was shown to elicit both NDV-specific and AMPV-C specific humoral immune responses, which substantially protected against challenge with the two virulent viruses [48]. This further demonstrates the incredible ability of NDV to serve as an efficient vaccine vector in turkeys.

In the geese industry, gosling plaque (otherwise known as goose hepatitis) accounts for the highest rates of mortality and mobility. The disease is caused by a goose parvovirus that mainly causes gastrointestinal disturbances due to the predilection of the virus for the intestinal wall [95]. Live and inactivated vaccines are available to control this disease in areas where it is endemic. However, the currently available vaccines have many setbacks that necessitate the need to develop alternative vaccines. Apart from the risk of reversion of live vaccines back to virulence, the cost of vaccines that are prepared in SPF geese allantoic fluid is outrageously high, owing to the scarcity of SPF geese or duck embryonated eggs. Thus, improved and more cost-effective vaccines are needed to adequately control this economically important disease of geese. Among the structural proteins of GPV, the VP3 contains the highest number of neutralizing epitopes, a property that qualifies it to be used as a suitable vaccine antigen against the virus. Recombinant genotype VII-based NDV expressing the VP3 of GPV developed as a bivalent vaccine was shown to simultaneously induce strong neutralizing antibodies against NDV and GPV in China [60]. Since the prevailing NDV strains in China for the past two decades have been genotype VII isolates, this chimeric NDV and GPV vaccine stand to offer excellent protection against the field NDV strains (because it is genotype-matched) and at the same time adequately protect against GPV. 

Other important avian pathogens against which NDV was used to deliver immunogens include infectious laryngotracheitis virus (ILTV). This pathogen primarily affects the upper respiratory system of chicken where it causes severe clinical signs such as dyspnoea, coughing and conjunctivitis, which inevitably lead to inappetence, reduced feed conversion efficiency, and a drastic decrease in egg production [96]. For a very long period of time, live attenuated vaccines have been widely used to control the disease. However, those vaccines have been shown to retain some level of residual virulence strong enough to cause clinical disease in chicken especially when administered as a spray [97]. Furthermore, they are associated with latent infection, which might later be reactivated especially in long-lived layers and breeders [98]. Thus, alternative vaccine platforms are required to optimally control this economically important disease of chicken. Deletion mutants of the virus devoid of glycoprotein J have been generated and shown to be highly protective in chicken [98]. Unfortunately, these vaccine candidates demonstrate poor in vitro replication kinetics. Attention therefore has shifted to recombinant fowl pox or HVT vectored vaccines that do not possess the risk of establishing latent infection. Again, these vaccines are only partially protective against ILTV [99]. Recently, a recombinant NDV LaSota strain engineered to express the glycoprotein D of the ILTV has been shown to induce a strong immune response that completely protected the vaccinated birds against the highly virulent ILTV challenge. Indeed, the level of neutralizing antibodies induced by the recombinant NDV vectored vaccine was even higher than that of commercial vaccines [55,100]. Given this superior humoral response and the stability of the virus, recombinant NDV expressing ILTV gD is a potential next generation vaccine candidate against ILTV infection in chicken.

### 4.3. Engineered NDV as a Vaccine Vector in Ruminant and Monogastric Animals

Middle East respiratory syndrome corona virus (MERS-COV) is the causative agent of a fatal human disease characterized by gastrointestinal and respiratory disorders [101]. The virus is harbored by bats and camels with the possibility of spill-over from these animals to man. Since camel–human contact in the Middle East is more frequent than bat–man interaction, the virus is more likely to be transmitted to man from camels, which were recently declared the major reservoirs of the virus in Saudi Arabia [102,103]. Hence, the most appropriate strategy to effectively control the disease is the development of a vaccine that will neutralize the virus in camel reservoirs. Recently, recombinant NDV Beaudette C strain was engineered to express MERS-CoV protein using reverse genetics approaches. When this chimeric virus was used to immunize camels in a prime-boost manner via the intramuscular route at the dose of 2 × 10^9^ EID_50_ per camel, neutralizing antibodies against MERS-CoV were elicited [50]. Although a challenge experiment was not performed in the above study due to the unavailability of large animal containment facility, the rise in the neutralizing antibodies titre, as determined by ELISA and virus neutralization tests, especially after the booster vaccination, suggests the potential immunoprotective property of the vaccine [50]. More so, the fact that NDV can easily be prepared in large quantities in embryonated chicken eggs makes this potential bivalent vaccine less expensive and therefore easily affordable by the less privileged farmers. NDV is therefore a potential vaccine delivery agent against infectious diseases in camels.

Bovine enzootic fever, otherwise known as 3-day sickness, is an acute febrile disease of cattle and buffalo caused by an arthropod-borne pathogen called bovine ephemeral fever virus (BEFV). Although the disease is rarely fatal, it causes huge economic loses in the form of drastic reduction of milk yield among the dairy cattle [104]. Various vaccine preparations ranging from live, inactivated to subunit vaccines are utilized to control this disease in different countries. However, despite the effectiveness of these vaccines, the immunity they induce is not long lasting [105]. This means that a booster vaccine must periodically be administered to maintain the antibody titres above the protective threshold in the immunized subjects. As an alternative, recombinant NDV vectoring BEFV G protein was constructed and used as a vaccine in cattle. After the initial and booster vaccination of 1-year-old cattle using the recombinant vaccine, a good neutralizing antibody titre was obtained [53], suggesting the potential of the recombinant virus to be used in the control of the disease. More importantly, the use of the vaccine will be highly relevant in disease eradication by making it possible to differentiate vaccinated from infected animals (DIVA).

Bovine herpes virus-1 is the causative agent of many diseases in cattle such as infectious pustular vulvovaginitis, balanoposthitis and infectious bovine rhinotracheitis. It is therefore an important pathogen that militates against cattle production in regions where it is endemic [106,107]. A number of vaccines are available in different preparations to control this virus. However, those vaccines are not without their shortcomings. The live vaccines, although highly immunogenic, may cause immunosuppression in the vaccinated host. On the other hand, the inactivated vaccines are very safe but poorly immunogenic [108]. The quest for more effective vaccines has led to the generation of recombinant NDV expressing the BHV-1 gD protein. This protein is among the three enveloped proteins of the virus that are involved in the initial infection and the induction of neutralizing immunity in the infected or vaccinated host. Interestingly, of the three envelope proteins, the gD protein has been shown to contain the most neutralizing T and B cell epitopes [109]. Little wonder, several recombinant vaccines against BHV-1 utilized the protein as an immunogen [110,111]. When the recombinant NDV expressing BHV-1 gD was used to immunize 3-month-old calves via the combined intranasal and intratracheal routes, BHV-1 specific IgA and IgG immune response were observed. Although the protection observed following challenge with the virulent BHV-1 was only partial [52], it was reasoned that a second dose of the vaccine might significantly improve the protective efficacy of this vaccine in cattle. 

Rift valley fever (RVF) is another important disease of ruminants with high zoonotic potential. It is characterized by abortion, foetal abnormalities and neonatal mortality especially in lambs [112]. To control the disease, formalin killed or live attenuated vaccines are often used. The killed vaccines are generally less immunogenic and so, adjuvants and booster vaccinations are required to enhance their effectiveness [113]. The live vaccines on the other hand are effective following a single administration but their safety in young animals is questionable. Hence, the current trend in the control of RVF is the development of cheap and effective vaccines that can be used across all age groups of animals [114]. In line with this requirement, many recombinant vaccines in the form of epitope-based synthetic peptides, plasmid DNA encoding the protein of interest, or viral vectors-based vaccines have been generated. Of these vaccines, the viral vectored vaccines seem to be the most useful tools for efficient delivery of RFV immunogens. A recombinant Venezuelan equine encephalitis virus expressing the RFV G2 protein was found to induce strong protective immunity in vaccinated mice [114]. However, this chimeric virus failed to grow to high titres in cell cultures, a problem that diminishes its potential as a vaccine. Interestingly, the recovery of a recombinant NDV expressing Gn protein of RVF as a potential vaccine was reported. When this vaccine was used to intramuscularly immunize calves at a dose of 2 × 10^7^ TCID_50_ per animal, a good level of anti RFV Gn neutralizing antibodies was observed. Although the serum neutralizing titres were only moderate, they were thought to be enough to protect against the virulent form of the disease [51]. With this level of immunogenicity added to the growth in high titres following inoculation in chicken embryonated eggs, NDV vectored RFV vaccine stands to be useful in the control of the disease. 

Recombinant NDV is also useful in the delivery of vaccine antigens in dogs and cats. Canine distemper, a severe life threatening viral infection of carnivores, is currently controlled using modified live vaccines whose safety is still questionable. Immunization with those vaccines has been reported to cause clinical disease due to reversion back to virulence [115,116]. Furthermore, pre-existing maternal immunity strongly interferes with the efficacy of these vaccines. Thus, a safer and more efficacious vaccine platform is needed to improve the current control strategy. One attractive strategy that could potentially overcome these challenges is the use of a recombinant viral vector system. Recently, a recombinant NDV expressing the H protein of CDV (rLaSota-CDV HN) was shown to elicit solid neutralizing immunity that protected against virulent CDV challenge in minks [67]. In view of the numerous advantages of the NDV vector, particularly its host restriction and non-pathogenicity in mammals, rLaSota-CDV stands to be a good alternative to the not-very-safe conventional live attenuated CDV vaccines.

Rabies is a highly fatal zoonotic viral disease of all warm-blooded animals associated with severe pathology in the nervous system. Humans predominantly get infected via bites from a rabid dog [117,118]. Thus, controlling the disease in dogs is crucial to its prevention in humans. Interestingly, both live attenuated and recombinant vectored vaccines are available for the control of rabies [119]. However, those vaccines possess some limitations that make the search for improved vaccines a continuous priority. For instance, most of the live attenuated rabies vaccines are not very safe and only induce suboptimal immune response in the vaccinated animals [119]. Furthermore, recombinant vaccinia virus vectored rabies vaccine has been linked to severe integumentary inflammation, and the vector may cause persistent infection in humans [120]. As a result, effective rabies disease control in both man and animals demands the development of an improved vaccine that overcomes those challenges. Using the LaSota strain as a backbone, a recombinant NDV expressing rabies virus glycoprotein (rLaSota-RVG) was constructed and recovered as a potential vaccine [59]. When used to vaccinate dogs and cats at a dose of 10^9.8^ EID50, the rLaSota-RVG induced a robust long lasting neutralizing immunity that effectively protected against challenge with a wild type rabies virus street strain.

Collectively, the NDV vector is not only an efficient vaccine vector in poultry, it has also been shown to be a promising vector in other animals such as cattle, camels, dogs, etc. Since NDV is apathogenic in non-avian species, efforts should be intensified towards its use as a vaccine vector in different animal species particularly against those diseases whose control strategies are confronted with a lot shortcomings and challenges. 

### 4.4. Engineered NDV as a Vaccine Vector in Man

Genetic manipulation of NDV has also been shown to be applicable in the delivery of human vaccines [111]. This is of particular relevance in the case of pathogens with serious biosafety concerns. For instance, Ebola virus, which causes a highly fatal disease in humans, can only be handled under BSL-4 facility because of its potential for human-to-human spread [121]. Therefore, conventional live attenuated Ebola vaccines are not likely to be safe due to the possibility of their reversion back to virulence. Moreover, inactivated Ebola vaccines that have been shown to be safe can only induce a suboptimal immune response that incompletely protects against lethal Ebola virus challenge [122]. Thus, there is the need for a safer and more effective vaccine platform. Among all the next generation Ebola vaccines, recombinant virus vectored vaccines appear to be the most promising. Rabies virus and Venezuelan equine encephalitis virus expressing Ebola GP are some of the recent vaccines that are currently under clinical trials in humans [123,124]. Recombinant NDV expressing Ebola GP has also been shown to stimulate strong neutralizing systemic and mucosal immunity following booster vaccination in monkeys [68,69]. This signifies the ability of NDV to effectively deliver Ebola virus immunogens as vaccines.

Successful delivery of HIV antigens has also been achieved using recombinant NDV vehicle [125]. Several live and inactivated vaccine candidates have been unsuccessful in the fight against the HIV pandemic, which continuously ravages the human population in different countries [126]. An ideal HIV vaccine should not only stimulate a long-lasting neutralizing antibody response, it should also elicit strong cell-mediated immunity, particularly a CD8^+^ response, which is believed to play a crucial role in virus clearance [127]. With the gag and pol proteins being the most important elicitors of robust immune responses against HIV [128], attempts have been made to construct a recombinant vectored vaccine expressing these proteins. In one study, genetically engineered NDV expressing HIV gp140 or gp160 have been shown to induce a strong anti-HIV humoral immune response following parenteral or intranasal administration in mice [61]. Similarly, recombinant NDV expressing HIV gag protein induced a strong cell-mediated immunity specifically directed against the gag antigen in mouse models. Indeed, co-immunisation of mice with recombinant NDV expressing HIV gag and another recombinant NDV expressing HIV envelope protein was shown to induce strong neutralizing antibody and cell-mediated immunity especially following intranasal vaccination [61]. 

The potential of NDV as a vaccine vector has also been evaluated against emerging human diseases such as Nipah virus encephalitis and severe acute respiratory syndrome (SARS). In one instance, NDV was engineered to express Nipah virus surface glycoproteins (F and G). When the engineered NDV was used to immunize mice at the dose of 10^8^EID50, a strong and long lasting Nipah virus-specific neutralizing immunity was generated [70]. Furthermore, recombinant mesogenic NDV engineered to express SARS-CoV spike S glycoprotein was found to not only induce a strongly protective immune response in mice, it also drastically reduced the viral load in the lungs, trachea and nasal turbinate following challenge with 10^6^TCID50 of SARS coronavirus [49]. Other human vaccines vectored by NDV have been extensively reviewed elsewhere [111].

## 5. Engineered NDV as an Improved Cancer Vaccine

### 5.1. Molecular Mechanisms of NDV Induced Oncolysis

As far back as seven decades ago, NDV has been recognized to replicate more efficiently in mammalian cancerous cells than in normal cells. This natural oncolytic tendency of NDV has been demonstrated both in cell culture systems and different animal models [129]. In fact, many oncolytic NDV strains are currently at various stages of clinical trials for use in humans. One of the possible mechanisms of this selective replication of NDV in cancerous cells is the differential activation of interferon signaling pathways in normal and cancerous cells. Normal human cells are equipped with the RIG-I receptor that efficiently senses the presence of NDV and elicits a measurable interferon response [130]. On the other hand, most human cancers have defective interferon pathways, and so they cannot easily antagonize the invasion and replication of NDV. Nevertheless, some NDV strains have been shown to efficiently replicate in cancers with an intact interferon antiviral system, suggesting the involvement of other mechanisms of NDV-induced oncolysis [131]. Previously, NDV has been shown to stimulate the upregulated expression of TNF-related apoptotic-inducing ligands (TRAIL) on monocytes leading to tremendous oncolytic effects in tumor cells bearing the TRAIL-R2 receptor [132]. Similarly, NDV has been shown to induce apoptosis in many cancer cell lines by triggering certain endoplasmic reticulum stress in a manner independent of P53 gene expression [133]. Thus, the oncolytic efficacy of NDV could also be related to its ability to stimulate both intrinsic and extrinsic apoptosis pathways. Other mechanisms of NDV-induced oncolysis include the upregulated expression of major histocompatibility molecules and cell adhesion receptors as well as the increased secretion of proinflammatory cytokines around the tumor site [134]. In summary, the strategies of NDV-mediated oncolysis can be broadly classified into those involving the manipulation of the cellular antiviral and anti-apoptotic pathways as well as other indirect mechanisms that involve the activation of innate and adaptive immune responses. 

### 5.2. Recombinant NDV as an Improved Oncolytic Agent

Despite the widely known oncolytic efficacy of wild type NDV, certain types of cancer are still resistant to the activity of the virus [134]. With the advent of reverse genetics, however, strategies of improving the oncolytic efficacy of the virus have emerged [135]. One of those strategies involves the manipulation of the F protein cleavage site of the virus. In one study, avirulent NDV was reported to have been engineered to encode polybasic instead of monobasic F cleavage site. When the recombinant virus was evaluated for anticancer activity, it was found to have a dramatically increased cell-to-cell fusion activity, replication kinetics, and tumoricidal effects in many human cancerous cell lines compared to the wild type virus [136]. This suggests an improved therapeutic potential of the virus. However, since NDV with a polybasic F cleavage site is potentially pathogenic in chicken, its usage as a cancer vaccine in humans is nowadays discouraged because of the fear of accidental spillage into the environment. Hence, the current focus is the generation of recombinant NDV that demonstrates excellent antitumor effects in humans while retaining its safety profile in chicken [137]. 

In line with the current research focus in NDV oncolytics, [138] generated a type-1 interferon sensitive avirulent NDV by abrogating its V protein expression. Although the recombinant virus failed to grow efficiently in normal cells due to interferon antagonism, it grew to a much higher titre and indeed induced apoptosis in many human cancerous cells with defective interferon signaling systems. Unfortunately, the oncolytic prospects of this recombinant virus are limited to only cancers with a non-functional interferon pathway since the virus is interferon sensitive. Therefore, in order to overcome this limitation, [135] created two major alterations in the genome of an avirulent NDV and recovered the recombinant virus using reverse genetics. These alterations are the modification of the F cleavage site from monobasic to polybasic and the insertion of influenza NS-1 gene into a lentogenic NDV backbone. While the creation of the polybasic cleavage site stands to improve cell-to-cell spread of the virus in cancerous cells, the influenza NS-1 protein through its potent anti-interferon and anti-apoptotic properties, allows the virus to evade the innate immune response following infection [136]. Thus, when the recombinant NDV expressing this protein was used to treat various cancerous cell lines, efficient replication, better syncytia formation, and enhanced oncolysis were noted especially among malignant melanoma cell lines. 

Other approaches used to enhance the oncolytic efficacy of NDV include reprogramming the virus to constitutively express certain interferons and proinflammatory cytokines [134]. A recombinant NDV expressing soluble IL-2 has been shown to effectively regress hepatocellular carcinoma in mice and lead to the establishment of strong immunity that completely protected the cured mice from future challenge with cancerous cells [139]. Furthermore, co-expression of IL-2 and IL-12 on the NDV backbone led to enhanced anti-hepatoma activity in mice. In another study, NDV was engineered to express IL-12 and or IL-2, and both recombinant viruses proved to be better anticancer agents than the wild type virus [140]. Furthermore, recombinant NDV strain AF2240 engineered to express IL-12 was found to be highly efficacious against human breast cancer [141]. In addition, [142] in a proof-of-concept study showed that recombinant NDV expressing GM-CSF demonstrated a superb oncolytic activity and an enhanced immunostimulation of innate immune cells compared to the wild type virus. Thus, although NDV is naturally oncolytic, reverse genetics technology can be used to improve its properties to overcome the potential limitations associated with the use of the wild type virus. Recently, recombinant NDV engineered to express IL-24 has been shown to demonstrate improved oncolytic efficacy in murine melanoma models [143]. A summary of the approaches used in enhancing the oncolytic potential of NDV using reverse genetics is shown in Table 2.

## 6. Concluding Remarks

For several decades, NDV was only known as a poultry pathogen [148,149] with some potential to treat human cancer [150]. However, with the discovery of reverse genetics, so many other prospects of the virus have been unearthed. Today, the virus is easily programmable into a protective bivalent vaccine against highly virulent NDV and other economically important poultry diseases. The virus can also be manipulated to generate rationally designed vaccines against several emerging infectious diseases of various domestic animals. Furthermore, the virus has demonstrated the potential to not only deliver vaccine antigens against fatal human diseases, but also serve as an improved oncolytic agent against a variety of human cancers. Thus, the impacts of engineered NDV in modern vaccinology are enormous. Given its simple genome, efficient replication, host restriction, and non-pathogenicity in most mammals, NDV is likely to be the vector of choice against many other emerging diseases of man and domestic animals.

## Author Contributions

M.B.B., A.R.O., K.Y. and A.H.J. provided the needed literature. M.B.B. wrote the manuscript. A.R.O., B.P.H.P., A.I., and M.H.-B. critically reviewed the manuscript. All authors have read and agreed to the published version of the manuscript.

## Figures and Tables

**Table 1:** NDV as an effective vaccine vector in various animal species.

Host	Disease	Immunogen	NDV Backbone	Type of Immunity	Reference
Monkeys	Severe acute respiratory syndrome	SARS-CoV S	Beaudette C	SARS-CoV S-specific CD8+ T cells	[49]
Camel	Middle east respiratory syndrome	MERS-CoV S	LaSota	Neutralizing antibodies	[50]
Cattle	Rift valley fever	RVFV Gn	LaSota	Neutralizing antibodies	[51]
Cattle	Infectious bovine rhinotracheitis	BHV-1 gD	LaSota	Neutralizing IgG and IgA	[52]
Cattle	Bovine ephemeral fever	BEFV G	Lasota	Neutralizing antibodies	[53]
Chicken	Infectious bronchitis	IBV S1 or full S	Lasota	CD4+ and CD8+ cells; Neutralizing antibodies	[42,54]
Chicken	Infectious laryngotracheitis	ILTV gD	Lasota	Neutralizing antibodies	[55]
Chicken	Highly pathogenic avian influenza	H5, H7, H9	Lasota	Neutralizing antibodies	[56,57]
Chicken	Infectious bursal disease	IBDV VP2	Lasota	-	[58]
Dogs and cats	Rabies	RV G	Lasota	Neutralizing antibodies	[59]
Goose	Gosling plaque	VP3 of goose parvovirus	NA-1	Neutralizing antibodies	[60]
Guinea pigs	Acquired immune deficiency syndrome	HIV gp160	Lasota	Neutralizing antibodies	[61]
Horse	West Nile fever	WNV *PrM/E*	Lasota	CD4+ and CD8+ cells; Neutralizing IgG	[62]
Mice	Acute pneumonia	RSV F	Hitchner B1	CD8+ cells	[63]
Mice	Nipah encephalitis	Nipah virus G and F	Lasota	T and B cells; Neutralizing antibodies	[64]
Mice	Vesicular stomatitis	VSV G	LaSota	Neutralizing antibodies	[65]
Mice	Viral gastroenteritis	NV VP1	LaSota and Beaudette C	CD8+ cells; Neutralizing antibodies	[66]
Minks	Canine distemper	CDV F and HN	LaSota	Neutralizing antibodies	[67]
Monkey	Ebola	EBOV GP	Beaudette C	CD8+ cells: virus specific IgA, and IgG	[68,69]
Pigs	Nipah encephalitis	Nipah virus G and F	LaSota	T and B cells; Neutralizing antibodies	[64]
Monkeys	Parainfluenza	HPIV3 HN	Beaudette C	Neutralizing antibodies	[70]
Turkey	Turkeys rhinotracheitis	F and G of AMPV type CG of AMPV type A and B	LaSota	Neutralizing antibodies	[48,71]

**Table 2:** Some strategies used to enhance the oncolytic efficacy of Newcastle disease virus.

	NDV Strain	Genetic Modification	In Vitro Effects	In Vivo Effects	Reference
1.	NDV Lasota	Change of F cleavage site from monobasic to polybasic	Enhanced oncolysis of neuroblastoma cells via intrinsic and extrinsic caspase independent pathways	Not done	[144]
2.	NDV Lasota	Expression of GM-CSF	Induction of strong interferon response in PBMC; Substantial tumor growth inhibition caused by vaccine cells modified with the virus	Not done	[142]
3.	NDV Hitchner B1	Modification of F cleavage site and insertion of influenza NS1 gene	Profound cytotoxicity on human myeloma cell line SKMel-2 and mouse melanoma cell line B16-F10	i. Infiltration of CD4 and CD8 positive cells ii. suppression of mouse footpad melanoma growth	[136]
4.	NDV Hitchner B1	Modification of F cleavage site and insertion of IL2	Not done	Complete colon cancer regression characterized by marked T cell infiltration in mice	[135]
5.	NDV-HUJ	Change of F cleavage site from polybasic to monobasic	Enhanced apoptosis of chemoresistant primary melanoma cells	Not done	[131]
6.	NDV Beaudette C	Truncation of V protein expression	Not done	Complete regression of duodenum adenocarcinoma in Balb/c mice	[138]
7.	Clone 30	Expression of IL2 and IL12	Enhanced tumor cell death on U251, HepG2, Hela, and A549 cells	Enhanced oncolytic effect on hepatocarcinoma in mouse	[140]
8.	NDV 73-T	Change of cleavage site from monobasic to polybasic; Insertion of 198 nucleotides at the HN-L junction	Enhanced oncolytic effect on CCD1125 and HT1080 cells	Inhibition of tumor growth in HT1080 xenograft mouse tumor model	[145]
9.	NDV MTH68	Expression of heavy and light chains of monoclonal antibody directed against Edb fibronectin antigen	Enhanced tumor selective cytotoxicity on HT 29 colon cancer cells	Not done	[146]
10.	FMW	Expression of chicken infectious anaemia virus proapoptotic protein	Enhanced killing of adenocarcinomic human alveolar cells	Significant regression of treated tumor	[147]
